# An abundance of aliC and aliD genes were identified in saliva using a novel multiplex qPCR to characterize group II non-encapsulated pneumococci with improved specificity

**DOI:** 10.1099/mic.0.001555

**Published:** 2025-04-25

**Authors:** Claire S. Laxton, Femke L. Toekiran, Tzu-Yi Lin, Beta D. Lomeda, Maikel S. Hislop, Lance Keller, Orchid M. Allicock, Anne L. Wyllie

**Affiliations:** 1 Department of Epidemiology of Microbial Diseases, Yale School of Public Health, New Haven, CT, USA; 2 Department of Cell and Molecular Biology, Center for Immunology and Microbial Research, University of Mississippi Medical Center, Jackson, MS, USA

**Keywords:** carriage, mitis-group streptococci, non-encapsulated pneumococcus, qPCR, saliva

## Abstract

Pneumococcal surveillance studies are reporting increasing prevalence of non-encapsulated pneumococci (NESp). NESp are an important reservoir for genetic exchange among streptococci, including for antimicrobial resistance, and are increasingly implicated in disease. Disease-associated NESp commonly carries the virulence genes *pspK*, or *aliC* and *aliD* in their *cps* locus instead of capsule genes. While molecular methods targeting the cps region are widely used for serotyping encapsulated strains, there are few assays available for the classification of NESp, meaning it is not widely undertaken. Therefore, we exploited these genes as targets for a novel qPCR assay for detecting and classifying NESp strains with improved efficiency and specificity. We conducted bioinformatic analysis on sequences from 402 NESp and 45 other mitis-group streptococci and developed a multiplex-qPCR, targeting *pspK*, *aliD* and two regions of *aliC*. The assay was validated using 16 previously identified NESp isolates. We then applied the assay to DNA extracted from culture-enriched saliva and isolated and characterized suspected NESp colonies, with confirmation by whole genome sequencing. Bioinformatic analyses demonstrated that previously published primers for *aliC* and *aliD* had low pneumococcal specificity but indicated that targeting two regions of *aliC* would improve species specificity, without compromising sensitivity. Our novel multiplex assay accurately typed all isolates. When screening saliva, we found a high prevalence of *aliC* and *aliD*, even in samples negative for pneumococcal genes *lytA* and *piaB*. Isolated colonies which were *aliC* and *aliD* positive could be differentiated as non-pneumococcal streptococci using our assay. Our multiplex-qPCR assay can be used to efficiently screen even highly polymicrobial samples, such as saliva, for NESp genes, to detect and differentiate potentially pathogenic NESp clades from closely related mitis-group streptococci. This will allow for a better understanding of the true prevalence of NESp and its impact on pneumococcal carriage and disease.

## Data availability

Whole genome sequences generated in this study were submitted to Genbank and accession numbers are listed in Table 1.

**Table 1.** Strains used in this study

Strain	Species	NCC, MLST	Source	**WGS accession**
**MNZ41**	NESp	NCC2b, 6153	Carriage isolate, South Korea [11]	ASJQ00000000 [9]
**MNZ85**	NESp	NCC2a, 2315	Carriage isolate, South Korea [11]	ASJF000030000 [9]
**MNZ37**	NESp	NCC1, 1106	Carriage isolate, South Korea [11]	ASJP00000000 [9]
**MNZ67**	NESp	NCC1, 1464	Carriage isolate, South Korea [11]	–
**MNZ49**	NESp	NCC2a, 448	Carriage isolate, South Korea [11]	–
**MNZ11**	NESp	NCC1, 6151	Carriage isolate, South Korea [11]	ASJW00000000 [9]
**B1351**	NESp	NCC1,	Conjunctivitis isolate, USA (Keller collection)	–
**E2_1300814**	NESp	NCC2, 1618^	Carriage isolate, Israel (Keller collection)	–
**E3_357**	NESp	NCC2, 344	Carriage isolate, Israel (Keller collection)	–
**E6_130069**	NESp	NCC2, 344	Carriage isolate, Israel (Keller collection)	–
**C144.66**	NESp	NCC1, 9570	Adenoiditis isolate, USA [58]	LSMB00000000.1 [58]
**D37**	NESp	NCC2, NF	Carriage isolate, Israel, SAMN13335414 [39]	JBJLPC000000000 (this study)
**D45**	NESp	NCC2, 344	Carriage isolate, Israel, SAMN13335426 [39]	JBJLPD000000000 (this study)
**D48**	NESp	NCC2, NF	Carriage isolate, Israel, SAMN13335438 [39]	JBJLPE000000000 (this study)
**H130**	NESp	NCC2, 4149	Pneumonia isolate, Hungary,SAMN13335479 [39]	JBJLPF000000000 (this study)
**H142**	NESp	NCC2, 344	Conjunctivitis isolate, Hungary,SAMN13335478 [39]	JBJLPG000000000 (this study)
**CL_5.50**	*S. mitis-like*	n/a	Carriage isolate, USA, this study	JBKOTR000000000
**CL_6.22**	*S. mitis-like*	n/a	Carriage isolate, USA, this study	JBKOTS000000000
**CL_6.35**	*S. mitis-like*	n/a	Carriage isolate, USA, this study	JBKOTT000000000
**CL_8.13**	*S. mitis-like*	n/a	Carriage isolate, USA, this study	JBKOTU000000000
**CL_9.43**	*S. infantis*	n/a	Carriage isolate, USA, this study	JBKOTV000000000

NESp = non-encapsulated *Streptococcus pneumoniae*; NCC = null capsule clade; MLST = multi-locus sequence type; NF = MLST not found; WGS = whole genome sequence. ^Double locus variant of ST1618 (aroE:2, gdh:98, gki:9, recP:new, spi:107, xpt:47 and ddl:new).

## Background

*Streptococcus pneumoniae* (pneumococcus) is an upper respiratory tract commensal bacterium, that following colonization, can cause invasive pneumococcal disease (IPD), particularly in vulnerable populations. Though pneumococcal conjugate vaccines (PCVs) contributed to a 50% reduction in pneumococcal-related deaths between 2010 and 2015, pneumococcus remains a leading cause of lower respiratory infection morbidity and mortality, globally [1]. PCVs target the major virulence factor, the polysaccharide capsule, of a limited number of the 106 known serotypes [2,3]. The use of PCVs has led to a general decrease in the prevalence of vaccine-targeted serotypes and has been effective in mitigating the outcomes of infection with these serotypes. However, both IPD incidence and carriage prevalence of non-vaccine serotypes have concomitantly increased [4,7].

Non-encapsulated *S. pneumoniae* (NESp) are strains that do not express a capsule, either due to transcription repression or loss-of-function mutations (Group I), or due to lack of capsule genes in the *cps* locus (Group II) [8]. Group II NESp are further subdivided into null capsule clades (NCCs) depending on the alternative genes they may harbour in the *cps* locus. NCC1 strains contain *pspK*, which encodes pneumococcal surface protein K (PspK) [9]. NCC2 strains contain *aliB*-like ORF1 and ORF2, otherwise known as *aliC* and *aliD*, respectively, which encode oligopeptide transporters AliC and AliD [10]. Based on the intergenic length polymorphism between a remnant *capN* gene and the flanking *aliA* gene, NCC2 strains are classified as either NCC2a or NCC2b [11]. Strains carrying only *aliD* have previously been categorized as NCC3; however, these are now generally considered to be closely related, non-pneumococcal mitis-group streptococci [12]. NCC4 strains have only transposable elements in the site of the chromosome where the *cps* locus is normally found [13].

Although NESp are mostly carried asymptomatically, they have been associated with conjunctivitis outbreaks, otitis media and cases of IPD [12,14,16]. The few instances of virulent NESp are often strains which harbour the NCC genes *pspK* or *aliC* and *aliD*, which may help to compensate for the lack of capsule. For example, PspK has been shown to increase adherence of NCC1 pneumococci to human epithelial cells *in vitro* and aids with colonization [9]. PspK also interacts with secretory IgA (sIgA) which decreases nasopharyngeal clearance and allows greater persistence on mucosal surfaces [9]. In addition, PspK has also been shown to increase transmission in an infant mouse model, which was exacerbated by influenza A infection [17]. Wajima *et al*. found that encapsulated pneumococci can naturally acquire *pspK* in place of their *cps* genes from neighbouring NESp [18]. This transformation may lead to non-encapsulation and increase their fitness *in vitro* [18]. For NCC2 strains, AliC and AliD regulate the expression of several genes, including choline-binding protein AC (CbpAC) which aids in reducing C3b deposition, and thus provide protection from classical complement-mediated clearance [19,20].

Without a capsule, NESp cannot be targeted by any existing PCV formulation. Therefore, vaccine-mediated pressure on the carriage of vaccine-serotype pneumococci has opened an environmental niche for NESp, which are also typically less susceptible to antibiotics than encapsulated strains, leading to an increase in their prevalence [21,24]. Though invasive NESp infections are currently rare, increased carriage prevalence may in turn lead to an increase in invasive infections. For example, a multidrug (including fluoroquinolone) resistant NCC1 NESp was recently isolated from a child with pneumonia [25]. There is also concern that NESp may transfer AMR or other virulence genes to encapsulated pneumococci, which could go on to cause invasive disease [26].

Molecular methods are now widely used to classify pneumococcal serotypes as part of carriage studies [27,32]. However, non-typeable strains are often not further classified when isolated [6,12, 26, 33]. This is partly because the conventional PCR (cPCR) assays most frequently used for NESp classification are laborious and their specificity for pneumococcus has not been empirically established [11,34]. This potential lack of specificity for NESp classification can be resolved by the use of a microarray assay which includes an extensive set of probes for pneumococcal capsular genes as well as *pspK*, *aliD* and *aliC*, and has been shown to be a highly effective method for serotyping, including for NCCs [30,35, 36]. Additionally, the ever-increasing accessibility of whole genome sequencing (WGS) allows for the highest level of species certainty and NCC classification [37]. This is further assisted by the development of tools such as SeroBA, which can resolve serotype from raw WGS reads using a k-mer approach, although it requires sequences from pure isolate samples [38]. Moreover, while potentially superior, both microarray and WGS approaches are costly and require a high level of user skill to execute and interpret, making them a less accessible option than qPCR.

Therefore, we developed a simple, pneumococcus-specific multiplex qPCR assay targeting the genes *pspK*, *aliC* and *aliD*, to allow accurate estimations of the prevalence of clinically relevant NESp strains, which can be deployed at scale for community screening and disease surveillance. The assay described in this study aims to fill the gap between simple but labour-intensive cPCR and more accurate but expensive molecular approaches, while balancing the need for sensitivity and specificity.

## Methods

### Bacterial strains and DNA extraction

Pneumococcal isolates (Table 1) were obtained from the Lance Keller and Larry McDaniel (University of Mississippi Medical Centre, USA), and Dan Weinberger, originally obtained from Ron Dagan (Ben-Gurion University, Israel), Adrienn Tóthpál and Eszter Kovacs (Semmelweis University, Hungary) [39].

Isolates were plated as a lawn onto Trypticase Soy Agar II (BD, USA) with 5% defibrinated sheep blood (Colorado Serum Company, USA) made in-house (BA plates) and incubated at 37 °C with 5% CO_2_ overnight. The lawn was harvested into 1 ml brain heart infusion (BHI) medium using a cotton swab and DNA was extracted from 200 µl of each sample using the MagMAX Viral/Pathogen nucleic acid isolation kit and a KingFisher Apex instrument (ThermoFisher Scientific) with modifications [40].

### Sequence analysis and qPCR assay design

NESp *cps* sequences from NCC1 (JF489996, JF489997, JF489998, KP762532, KP762534, KP762535, KP762536, KP762537) and NCC2 strains (MW205005, MW205004, MW205003, JF490007, JF490006, JF490005, JF490004, JF490003, JF490002, JF490001, JF490000, JF489999, AY653211, AY653210, AY653209) were obtained from the literature [11,21, 41, 42] and aligned using MAFFT to visualize conserved regions and create a consensus sequence for each NCC [43]. Primer3 (integrated into Benchling) was used to design primer and probe sequences, either based on previously designed primers [34], or targeting novel regions, which were then manually inspected using Benchling to assess secondary structure liabilities (shown in Table 2) and optimize for multiplex-qPCR [44,45]. Species specificity was verified *in silico* by running each primer and probe sequence, as well as the theoretical amplicon they produced (based on sequences from MNZ11 and MNZ85), through NCBI BLASTn [46]. Primers and probes (Table 2) were synthesized by Integrated DNA Technologies, Inc. (IA, USA), except those for the *lytA* and *piaB* dualplex, which were synthesized by Eurofins Genomics LLC (KY, USA).

**Table 2.** Primers and probes used in this study

Target	Sequence 5′−3′	ΔG homo-dimer	ΔG hetero-dimer	qPCR final concn	Source
**NESp multiplex**					
*pspK*	Forward: GCTTCATCTGCTGTAGAACAGG	−3.87	−2.28	500 nM	This study
	Reverse: TGACATCTGCCTTTTCTTGGAC	−1.28		500 nM	This study
	Probe: [HEX]-TGCTGAGGCAGAAAAAGATGTGAGCA-[IABKFQ]	−3.51		250 nM	This study
*aliC* (*aliC*1)	Forward: GACCAGATTACCAAGATCCAGCAAC	−1.63	−1.6	600 nM	[34]
	Reverse: GCCCTTTGTTATACCTAGATGTTTC	−1.46		600 nM	[34]
	Probe: [FAM]-AGATGCTAAGAAGGGTTCTGCC-[3IABKFQ]	−2.23		300 nM	This study
*aliC*(*aliC*2)	Forward: GCTCAAACTTTGAATCAACTAAAAAGG	−3.38	−3.19	600 nM	This study
	Reverse: CCTGACGGAAGTTCTTATTGAG	−2.88		600 nM	This study
	Probe: [ROX]-ATACGGCGATAAGATTGTCTATAGTCCACAAGAGG-[IABKFQ]	−5.03		300 nM	This study
*aliD*	Forward: CAAGGTGTGACTTTCCCAATTC	−1.18	−2.8	300 nM	Larry McDaniel
	Reverse: TGACTCAAGGGTCTGCTTAAC	−1.78		300 nM	Larry McDaniel
	Probe: [CY5]-TGGATGTGGCAGTTGATCAGACAAGT-[IABRQSP]	−4.13		150 nM	This study
***lytA*/*piaB* dualplex**					
*lytA*	Forward: ACGCAATCTAGCAGATGAAGCA			250 nM	[47]
	Reverse: TCGTGCGTTTTAATTCCAGCT			250 nM	[47]
	Probe: [FAM]-TGCCGAAAACGCTTGATACAGGGAG-[BHQ1]			250 nM	[47]
*piaB*	Forward: CATTGGTGGCTTAGTAAGTGCAA			250 nM	[48]
	Reverse: TACTAACACAAGTTCCTGATAAGGCAAGT			250 nM	[48]
	Probe: [HEX]-TGTAAGCGGAAAAGCAGGCCTTACCC-[BHQ2]			250 nM	[48]

ΔG for primer–dimer secondary structures were calculated by Primer3, using default settings and visualized with ViennaRNA [44,69]. The Gibbs free energy change (ΔG) is a measure of the spontaneity of the formation of a dimer between two same-sense (homodimer) or between sense and antisense (heterodimer) primers, displayed here in kcal/mol [70]. Sequences with values >−6.0 kcal/mol were used to avoid problematic secondary structures.

Newly designed primer and probe sequences for *aliC* (aliC2) were compared with those adapted from literature (aliC1) to predict their respective specificity for pneumococcus. The sequence for *aliC* covering both aliC1 and aliC2 primer binding regions from MNZ85 (JF490000, pos. 1000–2000) was searched using BLASTn of both the NCBI GenBank database and the Global Pneumococcal Sequencing project database. GPS sequences were retrieved using PathogenWatch, filtering for all sequences designated as Untypeable or Swiss-NT by SeroBA. Hits were downloaded and aligned to JF490000 : 1000–2000 using Geneious Prime 2025.0.3 (https://www.geneious.com). Duplicate entries and sequences which did not cover both primer binding regions were manually removed, and sequences were realigned using Clustal Omega v. 1.2.3. A consensus neighbour-joining tree was built using Geneious Prime 2025.0.3 tree builder (Jukes–Cantor distance model with majority greedy clustering consensus method, bootstrapping = 1000, minimum branch support = 80%).

### Strain characterization using qPCR

Following primer and probe concentration optimization, the NESp multiplex qPCR efficiencies was verified by standard curve using DNA extracted from MNZ11 (NCC1 reference, *pspK* positive) and MNZ85 (NCC2 reference, *aliC*/*aliD* positive). A dualplex TaqMan qPCR assay targeting the genes encoding the major pneumococcal autolysin LytA (*lytA*) [47] and pneumococcal iron uptake ABC transporter lipoprotein PiaB (*piaB*) [48,49] was also developed. The dualplex *lytA*/*piaB* efficiencies were similarly verified by standard curve using the MNZ85 DNA extract (*lytA/piaB* positive). DNA was quantified using a Qubit 4 Fluorometer (ThermoFisher Scientific) according to the manufacturer instructions and copy number per microlitre was calculated using the published genome size for each strain [9], assuming each base pair (bp) was 650 g/M [50]. Standard curves were run using 1 : 10 dilutions from 200 000 to 2 copies/µl (5 µl template input) and analysed with ThermoFisher DataConnect software and GraphPad Prism (iOS Version 10.2.1) to determine amplification efficiency and goodness of fit (*R*^2^), using linear regression and the equation: efficiency=10^(-1/slope)-1^.

The NESp multiplex was validated using DNA extracted from NESp and non-pneumococcal mitis-group strains, listed in Table 1. All qPCR assays were carried out in 20 µl reaction mixtures using 2X Luna Universal Probe Mastermix (New England Biolabs, MA, USA), 2.5 µl extracted genomic DNA (unless otherwise stated) and final concentrations of primers and probes according to Table 1. MNZ11 (*pspK* positive) or MNZ85 (*aliC* and *aliD* positive) were used as standards. All NESp assays were run on a QuantStudio 5 (Applied Biosystems) and all *lytA*/*piaB* assays were run on a BioRad CFX96, both under the following conditions: 95 °C for 3 min followed by 40 (NESp) or 45 (*lytA*/*piaB*) cycles of 98 °C for 15 s and 60 °C for 30 s.

qPCR amplification curves were manually inspected, and the run was designated as acceptable if the no-template control (water) and extraction-negative template control (DNA extraction of sterile BHI) Cq values were >40 and positive control Cq values were <30. Any amplification curves designated as false amplification were automatically assigned a Cq value of 41 and considered negative. Run data were exported and collated using Microsoft Excel (iOS Version 16.87) during which plate-to-plate variation was corrected by multiplying each sample Cq by an adjustment factor. The adjustment factor for each gene was calculated using reference Cqs derived from standard curves for each target, conducted in triplicate. For each experimental plate, the respective Cq from the reference standard curve (R) was divided by the Cq for the plate’s positive control (P) to generate a plate-specific ‘correction factor’ (CF), or CF=R/P. All test Cq values for that plate were then multiplied by the CF to give a final adjusted sample Cq.

Adjusted Cq data were visualized using GraphPad Prism (iOS Version 10.2.1). Samples were considered positive for any NCC gene when the respective adjusted Cq value was ≤35. Isolates with both *lytA* and *piaB* Cq values <40 and within 2 Cq of each other were classified as pneumococci. Isolates positive for *lytA* only were considered either *piaB*-negative NESp or other mitis-group streptococci [48,51], as further determined using WGS [48,51].

### Validation of the NESp quadruplex qPCR assay using saliva

#### Screening of saliva spiked with reference NESp

The ability to detect and recover NCC1 and NCC2 NESp strains from saliva was first tested by spiking 15 000 c.f.u./ml of each MNZ11 and MNZ85 into whole saliva which was collected by passive drooling from six healthy volunteers who were asymptomatic for respiratory illness and previously screened for the absence of *piaB* and *lytA*. Following spiking, each sample was culture-enriched within 10 min [32], harvested after overnight incubation and the DNA extracted as described previously [52]. Extracted DNA was tested using the NESp qPCR assay. Re-isolation of NCC-gene-positive colonies was attempted by thawing culture-enriched saliva samples (which had been stored at −80 °C) on ice, diluting 10 000-, 100 000- and 1 million-fold in BHI, and spreading 100 µl of each dilution onto BA plates. Plates were incubated overnight at 37 °C, 5% CO_2_. Following incubation, ~10 colonies per plate were picked and re-streaked onto fresh plates, which were incubated overnight as above [40]. Following incubation, a boilate of each colony was prepared for colony qPCR by touching a 1 µl sterile loop to the colony growth and transferring this to a thin-walled PCR tube containing 50 µl of sterile phosphate buffered saline and boiling for 10 min at 95 °C, followed by storage at 4 °C [40]; 2.5 µl of each boilate was added to the NESp qPCR assay. Colonies which tested positive for any NCC gene were subsequently tested for *lytA* and *piaB* to verify successful re-isolation of the original strains.

#### Saliva from pneumococcal carriage studies

DNA extracted from 70 remanent culture-enriched saliva samples, for which *lytA* and *piaB Cq* values had previously been determined [53,54], were screened to further validate the NESp assay. Isolation of NCC-gene-positive colonies was then attempted on a subset of 20 NESp-positive samples. Colonies which tested positive for any NCC gene were again subsequently tested for *lytA* and *piaB* and sequenced to confirm species.

#### WGS and species identification

Library preparation was undertaken using 50 ng of input DNA. Enzymatic fragmentation, end repair and dA-tailing were carried out according to the manufacturer’s instructions using Twist EF Library Prep 2.0 (Twist Bioscience Corp.). Next, unique molecular identifier (UMI) adapters with unique dual barcodes (Twist UMI Adapter System, Twist Bioscience Corp.) were ligated to the fragments and amplified using PCR. Amplified libraries were pooled equimolar, and quantity and fragment size were determined using Qubit (Thermo Fisher, Waltham, MA, USA) and D5000 ScreenTape System (Agilent, Santa Clara, CA, USA), respectively. Libraries were clustered, and approximately 1 million 150 bp paired-end reads were generated per sample, according to the manufacturer’s protocols at Yale Center for Genome Analysis using the NovaSeq6000 (Illumina Inc.).

#### Bioinformatic analysis

For WGS analysis, image analysis, base calling and quality check of sequence data were performed with the Illumina data analysis pipelines RTA3.4.4 and bcl2fastq v. 2.20 (Illumina). After quality pre-processing, reads for sequenced strains (CL_5.5, CL_6.22, CL_6.35, CL_8.13, CL_9.43, D37, D45, D48, H130, H142) were submitted to the comprehensive genome analysis service at PATRIC, assembled using Unicycler v. 0.4.8 and annotated using RAST tool kit (RASTtk) using genetic code 11 [55,56]. A phylogenetic tree with these genomes, plus reference genomes from representative streptococcal species, was constructed with PATRIC using RAxML v. 8.2.11, by aligning 100 genes using mafft, the JTTDCMUT protein model and RAxML Fast Bootstrapping (bootstrap=1000) [55,57]. Sequences were also deposited on NBCI Genbank and accessions are listed in Table 1.

## Results

### NESp sequence interrogation supported the design of a multiplex qPCR assay which was predicted to be both sensitive and specific for NESp NCC genes

Primers and probes targeting *pspK* were designed to target a similar locus of the gene as previously published assays [11]. A BLASTn search of the theoretical amplicon in the core_nt database was shown to be highly specific to *S. pneumoniae* (107/107 amplicon hits were sequences from *S. pneumoniae*, as of 26 November 2024). An *aliD* assay was designed to include a probe to work with existing *aliD*-specific primers [34]. However, a BLASTn (core_nt) search of the theoretical amplicon produced 51 hits with coverage and identity >95%, 34 of which were from *S. pneumoniae* and 17 of which were from non-pneumococcal streptococci (as of 26 November 2024). Given that *aliD* can be present in the absence of *aliC* in non-pneumococcal streptococci (historically categorized as NCC3) [12], it was determined that specificity to pneumococcus could be better derived from the *aliC* portion of the assay.

Primers and probes targeting two different *aliC* regions were designed, named *aliC*1 and *aliC*2. The first set, *aliC*1 included a new probe to work with existing *aliC* primers [34]. However, a BLASTn search of the *aliC*1 theoretical amplicon, as of 26 November 2024, produced 39 hits with coverage and identity >95%, 30 of which were pneumococcus, and 9 were non-pneumococcal streptococci. This suggested that both existing *aliC* and *aliD* PCR assays were not specific to pneumococcus and risked overestimating the prevalence of NCC2 NESp. The second set, *aliC*2, was designed to improve upon this lack of specificity by targeting a region ~600 bp upstream of the *aliC*1 primer–probe binding sites.

**Fig. 1.**
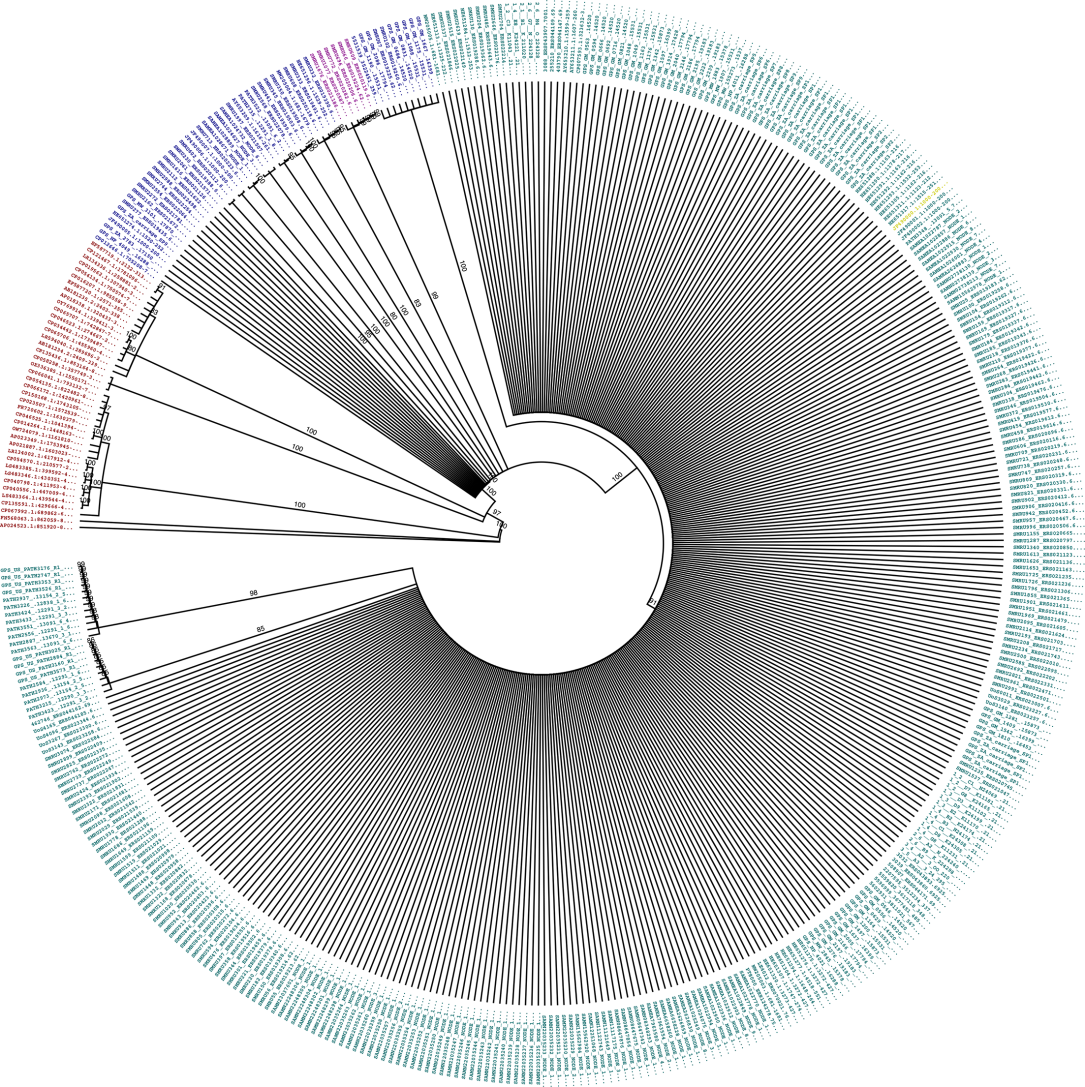
Phylogram of a 1000 bp region in *aliC* shows the primer binding regions for aliC1 has higher sensitivity and aliC2 has higher specificity for NESp. The 1000 bp sequence spanning the binding sites for all *aliC* primers was used in this study. Reference sequence (yellow) from JF490000 : 1000–2000 (MNZ85). Tips are coloured as follows: Turquoise: ≤2 SNPs in both aliC1 and aliC2 primer–probe binding sites (expected aliC1 and aliC2 qPCR positive) all sequences are from *S. pneumoniae*; Navy: ≤2 SNPs in aliC1 and ≥3 SNPS in aliC2 primer–probe binding sites (expected aliC1 qPCR positive but aliC2 qPCR negative) sequences are from a mix of *S. pneumoniae* and non-pneumococcal streptococci; Purple: ≤2 SNPs in aliC1 but at 3′-end of aliC1 probe, and ≥3 SNPs in aliC2 primer–probe binding sites (expected aliC1 qPCR high Cq or inconclusive, and aliC2 qPCR negative), all sequences are from *S. pneumoniae*; Red: ≥5 SNPs in both aliC1 and aliC2 primer–probe binding sites (expected aliC1 and aliC2 qPCR negative), all sequences are from non-pneumococcal streptococci. Nodes are labelled with WGS accessions, branch labels indicate consensus bootstrap support values over 80%. Branch length is proportional to evolutionary divergence.

To assess and compare the specificity and sensitivity of the *aliC*1 and *aliC*2 primer–probe sets, 447 *aliC* sequences were obtained by conducting BLASTn searches of a 1000 bp region from MNZ85 (JF490000 1000 : 2000), covering both primer binding sites against the NCBI core genome database (yielding 107 hits) and the GPS database of non-typeable strains (yielding 374 hits). Following removal of duplicates and low coverage hits, the remaining 447 sequences were aligned and a phylogenetic tree built (Fig. 1). These analyses revealed that 352/447 (79%) sequences were predicted to be amplified by both *aliC*1 and *aliC*2 assays due to having ≤2 SNPs across non-critical areas of their primer–probe binding regions. All 352 sequences were from *S. pneumoniae*. A further 48 (11%) sequences were predicted to be amplified by *aliC*1, but not *aliC*2, due to having ≥3 SNPs, including at critical locations in the *aliC*2 primer–probe binding regions such as the 3′-end. Of these, 44 were sequences from *S. pneumoniae*, and 4 from other streptococcal species such as *S. mitis*. An additional six pneumococcal sequences (1%) were predicted to have reduced amplification by *aliC*1, due to a SNP (C/A) in the 3′-most base of the aliC1 probe binding region, and no amplification by *aliC*2. The remaining 41 (9%) sequences had more than 5, and more often, over 20 SNPs across both primer–probe binding regions and thus were not likely to be amplified by either assay (Fig. 1). All 41 of these sequences were from strains classified as non-pneumococcal streptococci.

Therefore, *aliC*2 was predicted to detect *aliC* from pneumococci with 79% sensitivity and 100% specificity (352/402 true positives and 0/45 false positives) and *aliC*1 was predicted to detect pneumococci with 89–100% sensitivity and 89% specificity (396–402/402 true positives and 4/45 false positives). It was therefore predicted that inclusion of both *aliC*1 and *aliC*2 assays would yield a combined 90–100% sensitivity and specificity for NCC2 NESp, as well as provide some information on the prevalence of *aliC* homologues from non-pneumococcal streptococci.

### The NESp multiplex can be used to efficiently determine NESp NCC and identify NCC-gene-carrying non-pneumococcal streptococci

Following optimization, efficiencies of 93–102% were achieved for standard curve amplification of each of *aliC*1, *aliC*2 and *aliD* present in reference gDNA (Fig. S1, available in the online version of this article). However, the efficiency of the *pspK* assay was 83%, so absolute quantification for *pspK* in clinical samples should be inferred with some caution. Amplification efficiency of the dualplex *lytA* and *piaB* assay was 95–97% (Fig. S1).

**Fig. 2.**
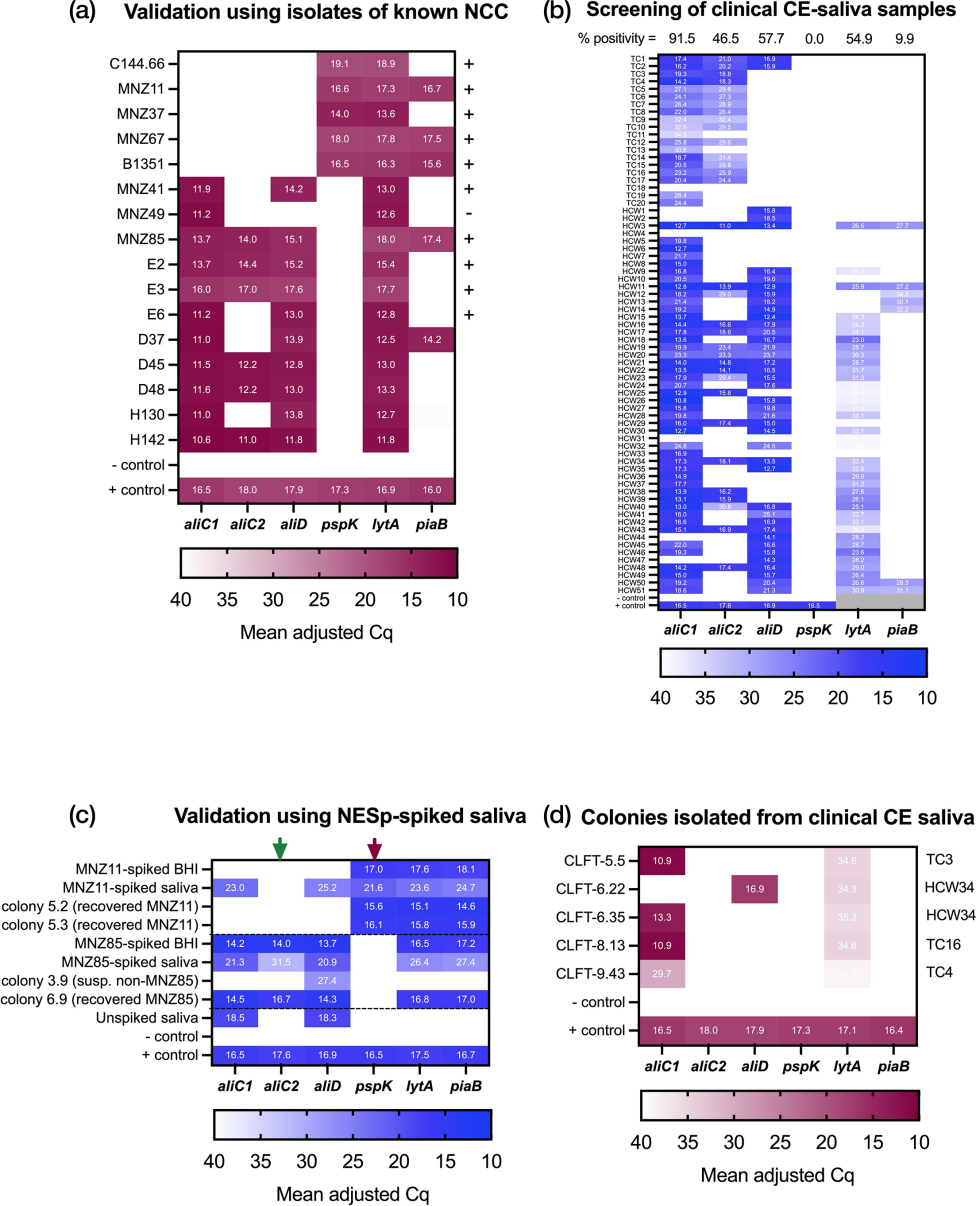
Heatmaps showing the detection of non-encapsulated *S. pneumoniae* (NESp) genes in bacterial isolates and culture-enriched saliva. Results suggest that aliC1 and aliD are near-ubiquitous in saliva and aliC2 more specifically detects pneumococci. Blue scale indicates results from DNA extracted from culture-enriched saliva, while the maroon scale indicates results from DNA extracted from single colonies. Positive (+) control in each was a mix of gDNA extracted from MNZ11 and MNZ85, concentration-normalized to 200 000 copies/µl, negative (−) control was nuclease-free water in at least technical duplicate. Panel (a): Mean-adjusted Cqs of NESp genes detected in DNA extracted from previously characterized, whole genome sequenced single colony isolates (test set; *n* = 2 technical). The plus sign on the right indicates that results align with previously observed results, or expected results based on sequencing, and minus sign indicates a discrepancy. Panel (b): Mean-adjusted Cq of NESp genes detected in DNA extracted from culture-enriched saliva spiked with 10 000 c.f.u./ml of either MNZ11 or MNZ85, indicated by dotted lines (*n* = 6 biological × 2 technical) or BHI (*n* = 3 biological × 2 technical) as well as respective colonies isolated from spiked culture-enriched saliva (*n* = 2 technical). Green and red arrows highlight the aliC2 and pspK columns, which can be used to indicate specificity to pneumococcus. Panel (c): Adjusted Cq of NESp genes detected in DNA extracted from culture-enriched (CE) saliva (*n* = 71×1 technical), along with previously determined lytA and piaB Cq values from historical carriage studies as a reference. Per cent (%) positivity indicates the percentage of samples designated as positive out of the total tested, according to a Cq cut-off of 35 for the NESp assay and 40 for the lytA/piaB assay. Grey boxes indicate missing lytA and piaB values due to the plate control data being unavailable from historical data, therefore Cq values for piaB and lytA were also not adjusted for plate–plate variation. Panel (d): Mean-adjusted Cqs of NESp genes detected in DNA extracted from single colonies isolated from culture-enriched saliva samples tested in panel c (*n* = 2 technical). Corresponding parent culture-enriched saliva sample ID is shown to the right.

The NESp assay was first validated using DNA extracted from 11 independently characterized single colony isolates (validation set) and 5 isolates previously designated as ‘non-typable pneumococci’ from our strain collection (test set), listed in Table 1. In all but one of the 11 validation set strains, the NESp assay results (Fig. 2a) agreed with previous findings [11,58]. The one discrepancy was in MNZ49, which was *aliD* positive in previous studies but negative here, which may be explained by each study using different primer sets, although the sequence for this strain is not available [11]. The *aliC*2 primer set had not previously been evaluated experimentally, but results from the validation set (Fig. 2a) were in agreement with sequence analysis of these strains where available (Fig. 1). For example, MNZ85 was *aliC*2-positive, and MNZ41 *aliC*2-negative, as expected, based on the presence of SNPs in the *aliC*2 primer binding regions of MNZ41.

Next, we validated the use of the NESp multiplex in saliva, in which mitis-group streptococci are known to be abundant [59,60], by spiking known quantities of MNZ11 (*pspK*+) and MNZ85 (*aliC*1, *aliC*2, *aliD*+) into *lytA* and *piaB* negative saliva samples, or BHI and testing using the *lytA/piaB* dualplex and NESp multiplex assays. We found that *aliC*1 and *aliD* were abundant in all saliva samples, including the unspiked control (Fig. 2b). However, *pspK* and *aliC*2 were only detected in the MNZ11 and MNZ85-spiked saliva, respectively. This suggested that we could expect to detect a high abundance of *aliC* and *aliD* in saliva, likely from other mitis-group streptococci present in the oral microbiota, but that our assay could specifically detect NESp-derived *pspK* and *aliC* (*aliC*2), and these could be used as pneumococci-specificity indicators.

When the NESp assay was applied to 71 culture-enriched saliva samples, we again found very high levels of *aliC*1 positivity (91.5%), as well as moderately high levels of *aliD* and *aliC*2 positivity (57.7 and 46.5%, respectively). We did not find any *pspK*-positive samples (Fig. 2c). Most, but not all, *aliD*-positive samples were also previously designated *lytA*-positive, although with relatively high *lytA* Cq values. Discrepancies in *lytA* and NCC gene Cqs may be partially explained by the lack of Cq adjustment in the *lytA* and *piaB* data in Fig. 2c, as it was collected as part of previous studies. However, in instances of large discrepancies (e.g. >5 Cq), it is more likely that much of the additional signal from *aliC* and *aliD* is being contributed from other *lytA* and *piaB* negative mitis-group strains also present in the sample.

**Fig. 3.**
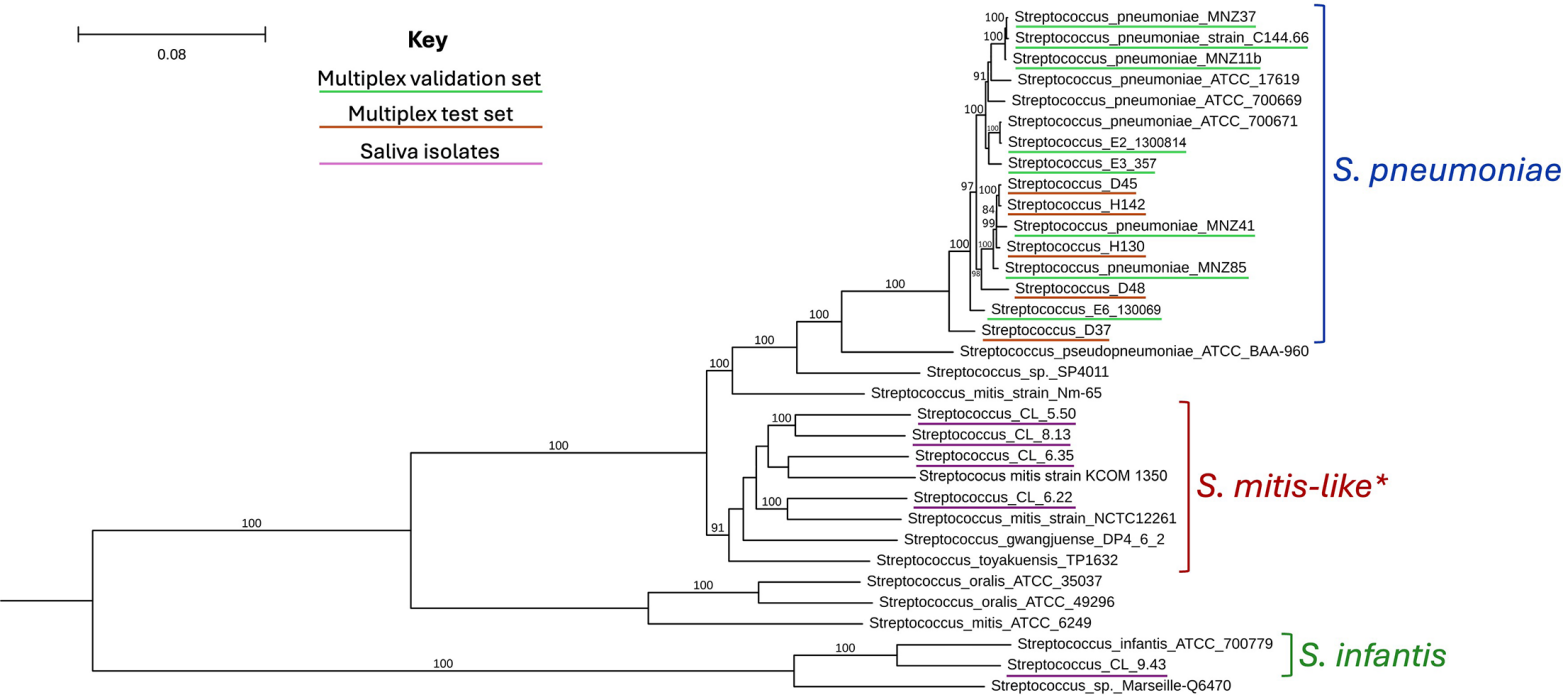
Phylogenetic tree containing whole genome sequences of isolates studied here, along with reference genomes of similar mitis-group streptococci. Study sequences are underlined as follows: orange: 8 independently characterized single colony isolates (validation set), green: 5 isolates previously designated as ‘non-typable pneumococci’ from our strain collection (test set), pink: 5 null-capsule clade gene-positive colonies isolated from culture-enriched saliva. Tips not underlined are reference sequences. Scale bar indicates genetic distance, branch labels indicate bootstrap support (values > 80% shown). *Clade labelled ‘S. mitis-like’ cannot be definitively classified due to low bootstrap support.

To better understand the source of the *aliC* and *aliD* signal in saliva, we attempted to re-isolate NCC-gene-positive colonies from the culture-enriched saliva samples. This was successful for 4/20 samples tested (one sample yielded two distinct colonies). When tested using qPCR, all five isolated colonies were *aliC*1 or *aliD* positive, but negative for *aliC*2 and *piaB* and only positive for *lytA* with high Cq values (Fig. 2d). This suggested they were non-pneumococcal streptococci. To confirm, the strains were sequenced and a phylogenetic tree (Fig. 3) was built using these sequences, along with those from our validation and test sets (Fig. 2a), and representative genomes from pneumococci and other mitis-group streptococci. Based on clustering in the phylogenetic tree, all five strains isolated from saliva in Fig. 2d were confirmed to be non-pneumococcal streptococci. This aligns with our prediction that non-pneumococcal streptococci could be distinguished from pneumococci via our new NESp multiplex assay, along with additional information from detection of *lytA* and *piaB*. The large Cq discrepancy between *aliC*/*aliD* and *lytA* in these non-pneumococcal strains may be explained by inefficient primer binding to *lytA* orthologues with low base-complementarity, which are known to be present in many non-pneumococcal streptococci [51].

## Discussion

Reports of a rise in the prevalence of NESp carrying *pspK*, *aliC* and *aliD* in the post-PCV era have prompted concern, due to the potential virulence of these strains and their propensity to acquire AMR genes [6,12, 21, 22, 26]. However, the most widely used assays for detecting these genes in NESp are either cPCR, microarray or WGS, which are each in different ways laborious and often require single colony isolation. Additionally, cPCR may lack specificity for pneumococcus [11], and microarray and WGS may be inaccessible in lower-resource settings. As a result, classification of NESp is often overlooked, and thus prevalence estimates may not be accurate. In this study, we developed a multiplex qPCR assay to assign NCC classifications to NESp, as well as distinguish between *aliC*-positive pneumococci and non-pneumococcal mitis-group streptococci, for application in polymicrobial samples such as saliva. Historically, the lack of *piaB* in many NESp strains has made it challenging to distinguish them from other mitis-group streptococci in pneumococcal carriage studies, as the latter can also contain *lytA* [48,49, 51, 61].

Previous studies have tackled this issue using a multipronged approach, verifying species using a combination of PCR testing for *lytA* and *piaB*, as well as *ply*—which this study did not target due to poor specificity [49]—MLST typing, and traditional culture methods such as optochin-sensitivity and bile solubility testing [42,62,64]. Subclassification of NCC by cPCR is frequently done as described by Park *et al*., using primers targeting *aliC* (with primers identical to our *aliC*1 assay), *aliD* and *pspK* [11]. Simões *et al*. streamlined this using a multiplex qPCR targeting *lytA*, *aliC* (equivalent to *aliC*1), 16S rRNA and *cpsA,* to improve the distinction between non-encapsulated pneumococci and other mitis-group streptococci [65]. However, as shown in Fig. 1, Fig. 2d and Fig. 3, targeting the *aliC1* loci of *aliC* may lead to the detection of non-pneumococcal streptococci such as *S. mitis* and *S. infantis*, which may also be *lytA-*positive, leading to false-positive calls for pneumococcus [51]. Inclusion of *piaB* has been adopted to more specifically distinguish pneumococci; however, as shown previously by Trzciński and colleagues, many NESp are *piaB* negative [48,49]. We corroborated this observation, finding here that 69% (11/16) of confirmed NESp strains were also *piaB* negative (Fig. 2d), reinforcing the assertion that reliance on *piaB* detection may lead to false negatives when detecting NESp. Another approach, taken by Yu *et al*., was to develop a multiplex platform combining PCR and monoclonal antibody assays, which also included *aliC (aliC1*), *aliD* and *pspK* genes. However, they had a limited number of NESp strains in their validation sets, and while simple, their approach required flow cytometric bead array technology, which may be less accessible to some labs [66].

Furthermore, some *aliC*/*aliD*-positive strains isolated from culture-enriched saliva in this study were also *lytA-*positive, albeit with relatively higher Cq values; however, all were identified as non-pneumococcal streptococci when sequenced. Thus, it is worth noting that without evaluating Cq differences, a simple positive–negative call could potentially lead to a false NCC2 NESp classification, especially when testing high-concentration DNA from isolates that may have some homology to pneumococcal *lytA* [51]. It is therefore critical to assess relative Cq value differences between all gene targets in a given sample. For example, it is advisable to require a Cq value range of <2 for all positive genes when making these calls based purely on qPCR data.

Our NESp multiplex assay can be applied to polymicrobial samples such as saliva, though the additional complexity of oral sample types indicates that NESp-suspected colonies should still ideally be isolated by culture and re-tested to make a definitive call. Still, this could be done with a simple colony PCR without the need for full DNA extraction [36]. Our study used remnant culture-enriched saliva samples to validate our assay and its specificity for pneumococci. The culture-enrichment step is used to reduce the presence of non-streptococcal species, while selecting for gentamicin-resistant species, such as mitis-group streptococci, from saliva [32]. Therefore, the addition of a culture-enrichment step can enhance the signal from these species carrying our target genes when present in the sample. Though not quantifiable using this method, the high proportion of culture-enriched saliva testing positive for *aliC* and *aliD*, from both adults and children, is worth remark.

However, although a larger than expected proportion of culture-enriched saliva tested positive for *aliC* and *aliD,* single NCC-positive colonies could οnly be isolated from 20% of NCC-positive samples tested, and all of these isolates were non-pneumococci streptococci. A possible explanation for this is that NCC2 pneumococci may be more fastidious than other streptococcal species. Indeed, we recently observed that NCC2 NESp exhibited a growth disadvantage during saliva culture enrichment compared with NCC1 NESp and encapsulated pneumococci, which may have hindered their isolation here [67]. A higher intensity screening method is needed to increase isolation success in the future, given the huge diversity of mitis-group streptococci present in culture-enriched saliva [59]. Additionally, it would be useful to further validate our assay on DNA extracted from raw saliva, as well as nasopharyngeal swabs, the dominant sample type utilized in pneumococcal carriage studies. We expect that, because of the reduced streptococcal species diversity present in nasopharyngeal swabs, this assay would perform even better than in saliva, and without the need for colony isolation.

The major limitations of this study are the poor success rate of NCC-positive isolation from culture-enriched saliva and the inclusion of a relatively small number of strains of limited geographic diversity for experimental validation. While further screening of a larger strain collection would strengthen these results, our *in silico* analysis of 447 streptococcal sequences from the NCBI and GPS databases supports our experimental conclusions that the use of dual-targeting of *aliC* can provide a high degree of sensitivity and specificity for NCC2 NESp isolates from multiple geographic regions.

In conclusion, the multiplex NESp qPCR assay described in this study offers a relatively simple and affordable method for screening for NCC genes, indicators of potentially virulent NESp strains, even in polymicrobial samples such as saliva, from which colony isolation is often difficult. Our assay targets two different regions of *aliC*, providing a high degree of sensitivity and specificity, especially when combined with *lytA* and *piaB* qPCR data. This assay can be easily deployed alongside existing molecular serotyping methods when typing strains as part of carriage studies, which will enable more thorough surveillance of increasingly clinically important NESp strains.

## Supplementary material

10.1099/mic.0.001555 Fig. S1.

## Abbreviations

CbpAC: choline-binding protein AC
cPCR: conventional PCR
IPD: invasive pneumococcal disease
MLST: multi-locus sequence type
NCC: null capsule clade
NESp: non-encapsulated pneumococci
PCV: pneumococcal conjugate vaccine
PspK: pneumococcal surface protein K
sIgA: secretory IgA
UMI: unique molecular identifier
WGS: whole genome sequencing
