# Supplementary material for: An abundance of aliC and aliD genes were identified in saliva using a novel multiplex qPCR to characterize group II non-encapsulated pneumococci with improved specificity
Source: Microbiology (Reading). 2025 Apr 25;171(4):001555. doi: 10.1099/mic.0.001555 (PMC12282236; doi:10.1099/mic.0.001555)
Supplement: Fig. S1. [file mic-171-01555-s001.pdf]

Supplementary data: An abundance of *aliC* and *aliD* genes were identified in saliva using a novel multiplex qPCR to characterize group II non-encapsulated pneumococci with improved specificity

Claire S. Laxton^, Femke L. Toekiran, Tzu-Yi Lin, Beta D. Lomeda, Maikel S. Hislop, Lance Keller, Orchid M. Allicock, Anne L. Wyllie^
^claire.laxton@yale.edu; ^awyllie@gmail.com

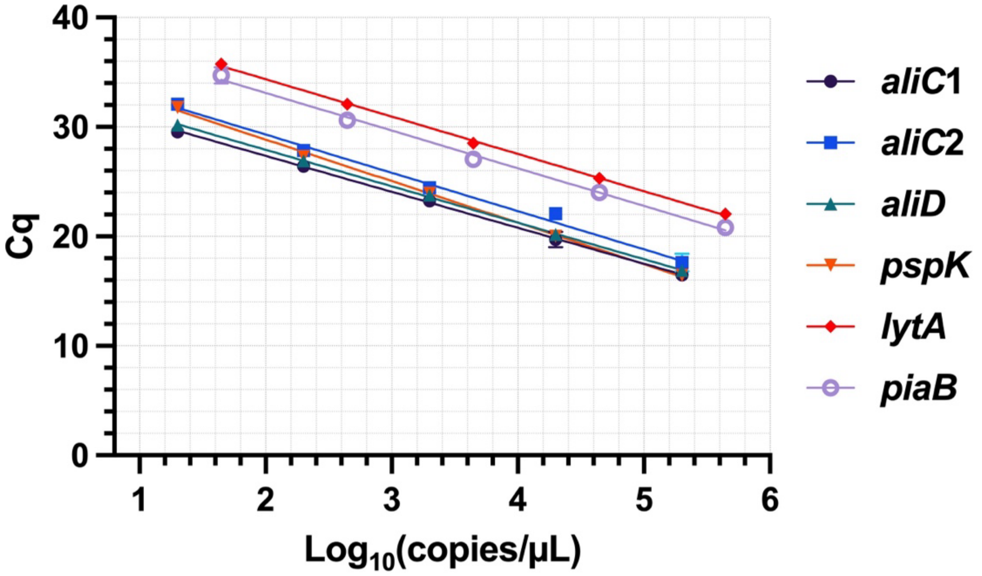

Target	Equation	Rsquared	Efficiency
aliC1	Y = -3.285*X + 33.93	0.9959	101.56%
aliC2	Y = -3.496*X + 36.30	0.9889	93.22%
aliD	Y = -3.333*X + 34.58	0.9988	99.54%
pspK	Y = -3.796*X + 36.44	0.9977	83.42%
lytA	Y = -3.411*X + 41.18	0.9982	96.41%
piaB	Y = -3.443*X + 40.00	0.9926	95.18%

Supp. Figure 1: Standard curve for the NESp multiplex qPCR assay. *Ten-fold serial dilutions of gDNA extracted from S. pneumoniae strains MNZ11 (positive for* pspK*) and MNZ85 (positive for* aliC1, aliC2, aliD, lytA *and* piaB*) were run in triplicate (mean + SD plotted). Quantity is shown in* Log_{10}*(copies/μL) from 5 μL input/reaction. Linear regression statistics and inferred amplification efficiencies are shown in the table below the graph.*
